# *In Silico* Assessment of Potential Druggable Pockets on the Surface of α_1_-Antitrypsin Conformers

**DOI:** 10.1371/journal.pone.0036612

**Published:** 2012-05-08

**Authors:** Anathe O. M. Patschull, Bibek Gooptu, Paul Ashford, Tina Daviter, Irene Nobeli

**Affiliations:** 1 Department of Biological Sciences, Institute of Structural and Molecular Biology, Birkbeck, University of London, London, United Kingdom; 2 ISMB Biophysics Centre, School of Science, Crystallography, Birkbeck, University of London, London, United Kingdom; Bioinformatics Institute, Singapore

## Abstract

The search for druggable pockets on the surface of a protein is often performed on a single conformer, treated as a rigid body. Transient druggable pockets may be missed in this approach. Here, we describe a methodology for systematic *in silico* analysis of surface clefts across multiple conformers of the metastable protein α_1_-antitrypsin (A1AT). Pathological mutations disturb the conformational landscape of A1AT, triggering polymerisation that leads to emphysema and hepatic cirrhosis. Computational screens for small molecule inhibitors of polymerisation have generally focused on one major druggable site visible in all crystal structures of native A1AT. In an alternative approach, we scan all surface clefts observed in crystal structures of A1AT and in 100 computationally produced conformers, mimicking the native solution ensemble. We assess the persistence, variability and druggability of these pockets. Finally, we employ molecular docking using publicly available libraries of small molecules to explore scaffold preferences for each site. Our approach identifies a number of novel target sites for drug design. In particular one transient site shows favourable characteristics for druggability due to high enclosure and hydrophobicity. Hits against this and other druggable sites achieve docking scores corresponding to a K_d_ in the µM–nM range, comparing favourably with a recently identified promising lead. Preliminary ThermoFluor studies support the docking predictions. In conclusion, our strategy shows considerable promise compared with the conventional single pocket/single conformer approach to *in silico* screening. Our best-scoring ligands warrant further experimental investigation.

## Introduction

The desire to modulate protein function with small molecules that can be administered as drugs has led to a plethora of studies attempting to define and calculate the “druggability” of sites on a protein [1], [2], [3], [4], [5]. Most studies have relied on experience from inhibiting enzymes acting on small molecule substrates. Here the target sites are well-formed surface pockets, characterized by high curvature and low solvent accessibility. Recently “harder” targets have been addressed. These include protein-protein interactions and proteins belonging to large homologous superfamilies e.g. kinases. In the former, the interfaces are larger and flatter [6]. In the latter, inhibiting the common active site risks serious cross-class side effects. Both these issues may be addressed by targeting clefts that are not necessarily associated directly with the protein’s biochemical function. The idea is that binding of small molecules to such clefts may be more favourable and could still allosterically modulate protein function, e.g. via preferential stabilization of a particular state within the conformational landscape of the protein in solution.

The search for suitable allosteric clefts requires consideration of functional relevance and druggability. Functional relevance is usually less obvious from structural snapshots for an allosteric site than an active site. It may be deduced experimentally by mutagenesis, or through observation of the binding site of known ligands. Druggability has traditionally been indirectly assessed by computational studies (docking) or *in vitro* screening. More recently, quantitative predictors of cleft druggability have been devised [2], [3], [5], [7], [8], [9]. These commonly assess the size, shape, buriedness and hydrophobic character of a site. However, a major limitation is currently not addressed routinely: the transient character of some clefts that may otherwise be of interest in drug design. Druggable pockets on a protein’s surface are most commonly assessed using a single 3D structure. This is unsatisfactory because proteins undergo dynamic changes in solution, sampling multiple conformations, each with potentially different surface pockets. The existence of multiple conformers is especially relevant to ligand recognition. Ligand binding inherently tends to conformational selection [10], [11], a process by which protein-ligand interactions lower the free energy of a conformer, increasing the stability and population of a state that may otherwise rarely be observed. In recent years some notable efforts have been made to identify transient sites. In the approach pioneered by Eyrisch and Helms [12], trajectory snapshots from molecular dynamics simulations revealed transient pockets on the surfaces involved in protein-protein interactions. In a more recent study, the same authors showed that transient pockets could also be revealed by methods that were more efficient computationally than molecular dynamics, albeit usually at the cost of reduced pocket diversity [13]. In another interesting study, Schmidtke *et al*. [14] demonstrated how pocket tracking across multiple structures with the program fpocket can highlight important changes to a pocket, arising from both dynamics of a single protein as well as evolutionary time in a family of homologues. Recently, the importance of employing multiple protein conformers in virtual screening has been highlighted [15], [16], [17] and there is a growing trend for incorporating notions of flexibility in the docking process. Despite these pioneering studies, most current *in silico* screening starts with the selection of a single pocket from a single conformer.

**Figure 1:**
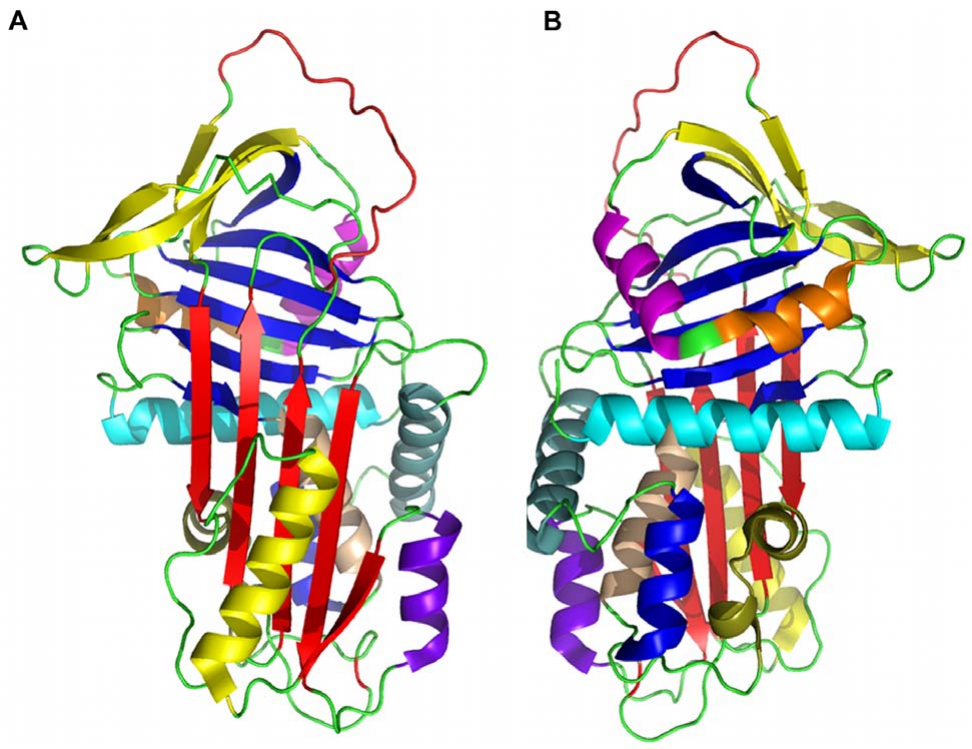
The structure of the wild type α_1_-antitrypsin. Front (A) and back (B) views of the structure of A1AT in cartoon representation (PDB entry: 1qlp). The secondary elements are coloured as follows. ß-sheets: A (red), B (blue), and C (yellow); helices: A (cyan), B (apricot), C (blue), D (grey-green), E (purple), F (yellow), G (orange), H (pink), I (olive); loops: reactive centre loop (RCL, red), all other loops (green).

In this study we demonstrate how the approach of employing multiple protein conformers at the selection-of-pocket stage can be combined with predictions of druggability, to aid the identification of transient, novel druggable pockets often missed in single conformer approaches. Our study focuses upon α_1_-antitrypsin (A1AT), the archetypal member of the serpin (serine protease inhibitor) superfamily [18]. Its characteristic native fold (Figure 1) is metastable and this is key to its antiprotease function [19]. It is an excellent candidate to assess our strategy for a number of reasons. Firstly, A1AT is a medically important target. Its metastability is subverted by pathogenic mutations that cause A1AT to polymerise. This causes diseases of the liver (neonatal hepatitis, cirrhosis and hepatocellular carcinoma) and lung (early-onset emphysema) through loss- and gain-of-function mechanisms [20]. Secondly, the biological function and dysfunction of serpins is coupled to marked conformational changes involving large rearrangements of their structure [21]. Moreover, extensive mutagenesis experiments demonstrate that mutations around surface clefts can significantly alter the stability of native A1AT [22]. Metastability is therefore related to pocket vacancy, indicating that ligand binding in a range of allosteric sites may modulate stability, and hence, pathological conformational change. Lastly, a range of high-resolution crystallographic datasets are available for wild type and mutant A1AT species in native, metastable (or stressed ‘S’) and relaxed (‘R’), hyperstable states, allowing comparison of computationally derived conformers with structural data. Following docking studies we have gone on to assess some of our most promising findings experimentally, identifying small molecule ligands with potential for development as novel therapeutics.

## Results

### Identification of Surface Pockets Present in Crystal Structures of α_1_-antitrypsin

**Figure 2:**
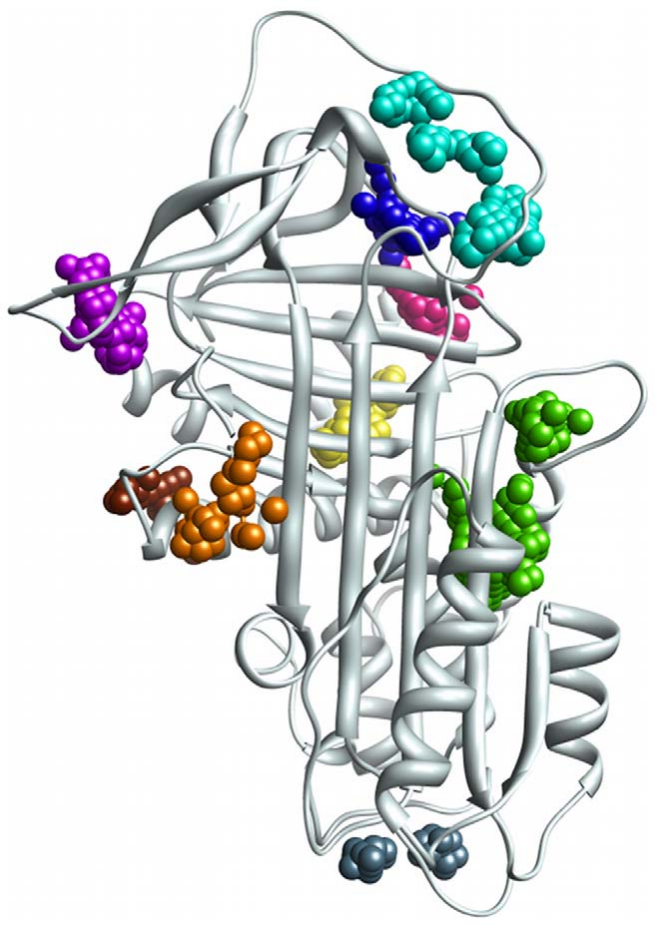
The nine top-ranking surface pockets identified by SiteMap on α_1_-antitrypsin. Coloured spheres represent the SiteMap predictions for eight top-ranking surface clefts on the wild type α_1_-antitrypsin (PDB entry 1qlp, in grey cartoon representation): site A: green, B: cyan, C: blue, D: purple, E: fuchsia, F: orange, G: slate blue, H: brown. The yellow spheres correspond to the ninth site, I, a cleft identified on crystal structures of A1AT containing the Ala70Gly mutation.

We denote the eight top-ranking surface clefts identified by SiteMap [2] on the structure of native A1AT (PDB entry: 1qlp) A to H (Figure 2). Sites A, D and G are each clearly distinct from other cavities, whereas sites C, B and E, as well as F and H are very close in space. The B, D and G sites are all defined by loop regions. In the case of site B, the loop involved is the reactive centre loop (RCL). It is also interesting to note that sites C and E are proximal to the glycosylation site Asn247, whereas D is proximal to glycosylation site Asn46. The largest predicted site on the native wild type A1AT (1qlp) is site A, adjacent to strand 2 of β-sheet A. This site scores the highest for tight binding of drug-like ligands with SiteMap scores (SiteScore 1.03, Dscore 1.03) highly consistent with those observed in sites binding drugs with a submicromolar K_d_ (mean 1.01) [2].

**Figure 3:**
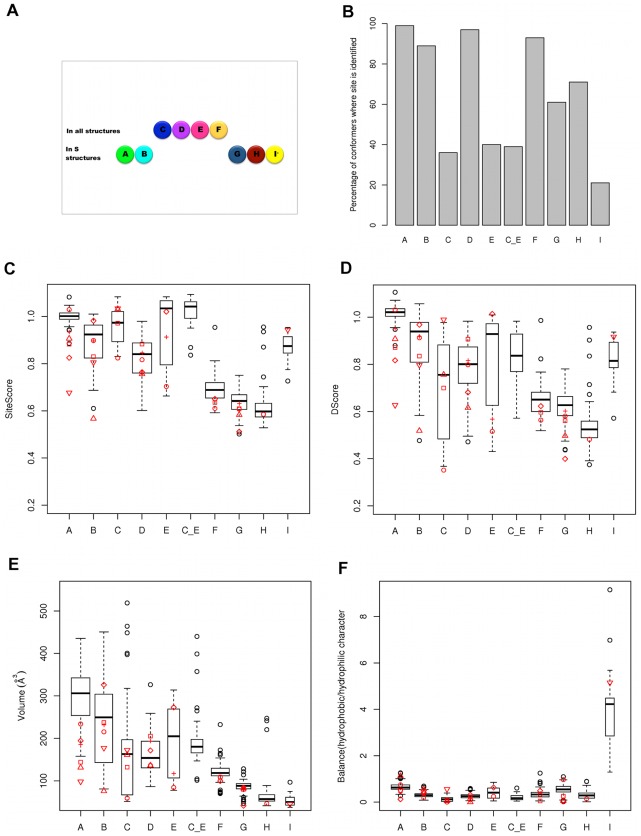
Properties of surface pockets in crystal structures and *in silico* conformers of α_1_-antitrypsin. Persistence of clefts A–I among A1AT crystal structures (A) and computationally produced conformers (B). Where the sites C and E overlapped, the data are presented under the label “C_E”. The distribution of SiteMap calculated properties for the 100 *in silico* conformers are shown as boxplots: SiteScore (C), DScore (D), site volume (E) and hydrophobic vs. hydrophilic character balance (F). The corresponding data for crystal structures are shown as red symbols superimposed on the boxplots; 1qlp (circle), 2qug (plus sign), 3cwm (square), 1hp7 (diamond), 3drm (triangle point up), 1oph (triangle point down). Data are shown only for sites identified within PDB entries for native (stressed, ‘S’) forms of A1AT, as these are likely to be the appropriate target states for the design of polymerization inhibitors.

Having identified potentially interesting sites on a single crystal structure, we assessed how persistent these sites were across our dataset of different crystal structures of A1AT (Table 1 and Figure 3A). In structures containing the metastabilizing mutation Ala70Gly (PDB entries: 1hp7, 1oph and 1iz2), we identified an additional site (here referred to as site “I”), located between the H-helix, the s4-s6 of the B ß-sheet and the A-helix. This site is small (45****Å^3^), and very hydrophobic (the ratio of hydrophobic to hydrophilic character measured by SiteMap’s “balance” property is 5.1, with 1.6 being the average balance for tight-binding sites [2]). Despite the small size of this site, the corresponding Site- and Dscores (0.92 and 0.92 respectively) calculated by SiteMap indicate a promising pocket for targeting with small molecule drug-like ligands. Although site I is present as a cavity in the remaining non-mutated structures, it is not solvent-accessible, and so is not identified by SiteMap. Interestingly, in PDB entry 1hp7, sites E and I are combined by SiteMap into one site, indicating that a ligand could possibly straddle both. Of the remaining eight sites, four are present in both the stressed and relaxed forms of A1AT (C, D, E, F), and four are only found in the stressed form (A, B, G and H). We summarise our results for the properties of each site in Figure 3.

**Table 1:** The dataset of selected crystal structures of A1AT used in this study.

Description	PDB id	Resolution (Å)	Mutations
Stressed – Native wild type	1qlp [30]	2.00	None
Stressed – Native wild type	2qug [41]	2.00	None
Stressed – Native with citrate bound	3cwm [41]	2.51	None
Stressed – Native mutant	1hp7 [42]	2.10	Ala70Gly
Stressed – Native mutant	3drm [32]	2.20	Thr114Phe
Stressed – Native mutant	1oph [43]	2.30	Ph351Leu, Thr59Ala, Thr68Ala, Ala70Gly, Cys232Ser, Met358Arg, Met374Ile, Ser381Ala, Lys387Arg
Relaxed – Uncleaved RCL (kinetic trap)	1iz2 [37]	2.20	Phe51Leu, Thr59Ala, Thr68Ala, Ala70Gly, Arg101His, Val364Ala, Met374Ile, Glu376Asp, Ser381Ala, Lys387Arg
Relaxed – Cleaved reactive loop	1ezx [19]	2.60	None

A large variation is observed in the volumes of all the larger sites among the eight crystal structures studied reflecting significant conformational changes across this dataset. However, even the largest of these sites (1qlp, site A: 234 Å^3^) is small compared with the average volume of drug-binding sites (reported as 600 to 900 Å^3^, depending on the method used to measure them [23]). Nevertheless, six of the sites (A, B, C, D, E and I) have a median SiteScore higher than 0.8, the recommended value for distinguishing drug-binding from non-drug binding sites [2]. Sites A, C, and E demonstrate SiteScores >1.01, consistent with submicromolar drug-binding, in at least one crystal structure [2].

### Incidence and Variability of Surface Pockets within a Computationally-generated Conformer Ensemble

An ensemble of 100 A1AT conformations was generated from the native wild type structure 1qlp using the distance constraints-based method within CONCOORD [24] (Figure S1). SiteMap was then used to assess pockets A–I across the entire computationally generated native-like ensemble. The frequency of occurrence of each site across all conformers is summarised in Figure 3B. The boxplots in Figures 3C–F summarise selected SiteMap property results for these sites. Similar trends for the volumes and site scores are observed for conformations produced using more extensive sampling, or a different structure of native wild type A1AT (2qug) as the starting point for the CONCOORD simulation (data not shown). The majority of the values for the Site- and DScores (Figures 3C and 3D respectively), volume (Figure 3E), and hydrophobic/philic balance (Figure 3F) for pockets in the crystal structures are within the boxplot limits. Thus the A1AT cavity characteristics explored by the computational conformers are supported by crystallographic observations. In addition, more detailed assessment of the computational conformers demonstrating the maximum Dscore for each cavity using the PROSESS server [25] indicated they were of comparable quality to experimental structures in our dataset in terms of geometry and packing (Table S1).

The behaviour of the A site across the computationally generated conformeric ensemble demonstrates the conservative nature of the conformational lability simulated by the program CONCOORD. Within the dataset of crystal structures of native A1AT the A site is largest and most druggable in 1qlp, the starting template for our CONCOORD simulation. The site is retained in 96% of the generated ensemble (Figure 3B) and displays higher volumes (Figure 3E) and druggability scores (Figures 3C & D) across these conformers than observed across the crystallographic structures. Despite this conservative approach, the ensemble generated by CONCOORD demonstrates that even these small fluctuations can have major consequences for surface clefts in A1AT, simulating pocket “breathing” in solution. Thus pocket volumes varied ≤3-fold for many sites (Figure 3E) and druggability scores showed up to 2-fold variation (Figure 3D). For many sites a source of high variability was their merging with other sites via formation of a channel of interconnected subsites. In particular, a channel ran from the RCL to the H-helix incorporating sites B, C, E and I in various combinations across several conformers (Figure S2).

A number of other sites have the potential to achieve druggability scores comparable to site A within the ensemble. However, the spread of scores across the conformational ensemble (Figure 3C) indicates that the ligand-favouring properties of these sites are subject to greater fluctuation than the A site. Only three sites (F, G and H) have median SiteScores below the 0.8 recommended cut-off for promising drug targets. In general, the SiteScore for a pocket correlates with the volume of that pocket, but it is interesting that site I, although relatively small, scores very highly (its median druggability score is highest after site A, among sites not defined by the RCL). This is probably due to its strongly hydrophobic environment (see Figure 3F), which has highly favourable drug binding characteristics.

### Surface Cleft Variability Assessed by Provar

**Figure 4:**
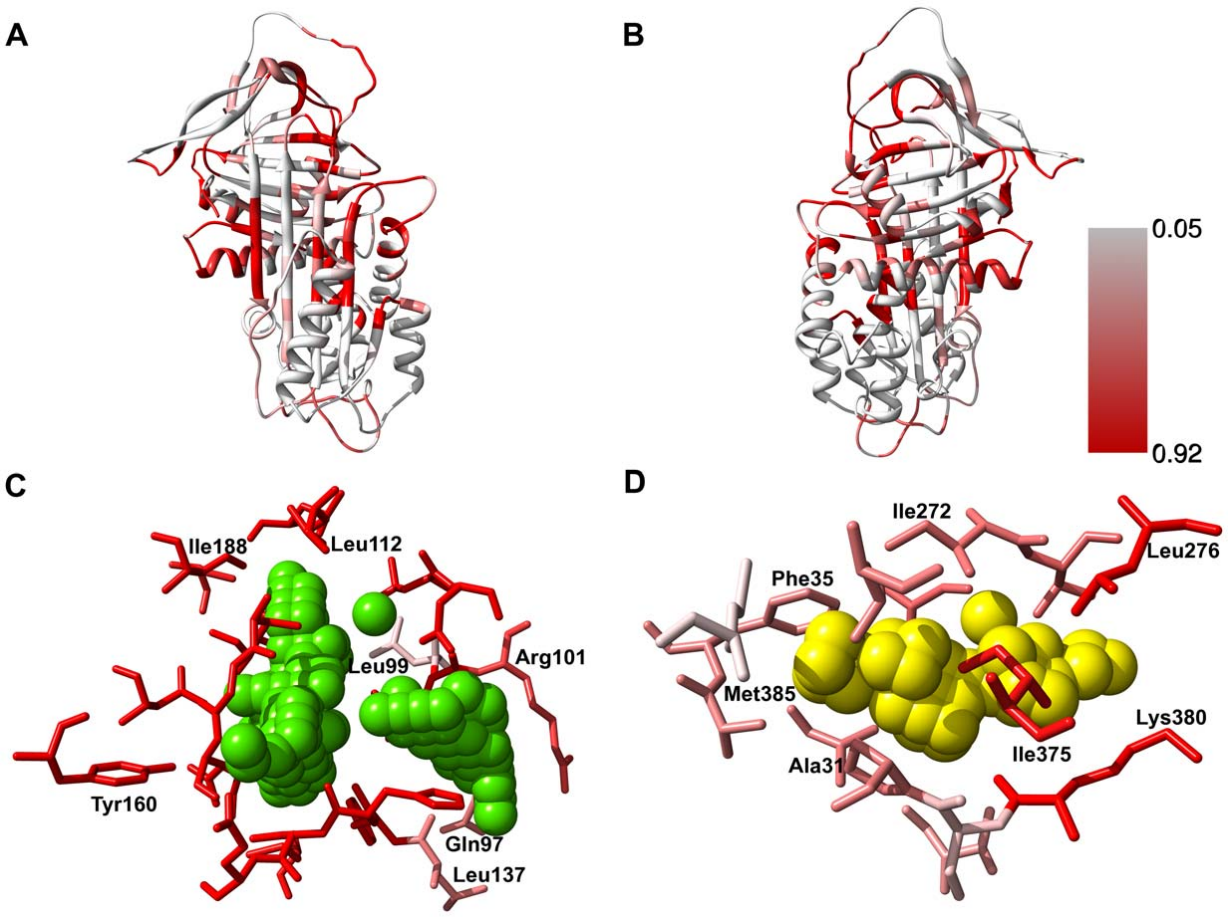
The pocket-lining propensity of the residues of α_1_-antitrypsin calculated with Provar. Ribbon representation of A1AT (front, A and back, B) coloured by the residue-based Provar probabilities. Provar colours each protein residue according to its probability of being pocket-lining in an ensemble of conformers (here, 100 CONCOORD-produced conformations of A1AT). The first (0.05) and third quartile (0.92) of the probability distribution are used as the white and red limits of the spectrum respectively. Hence, residues appearing red belong to the top quartile distribution, i.e., in this case, they are pocket-lining in more than 92% of the conformers. (C) and (D): The SiteMap predictions for two pockets (A and I respectively) are shown as solid spheres, and every residue with an atom within 3.75****Å of any sphere is shown in stick representation coloured by its Provar value. Depth-cueing has been switched off in these figures to preserve the variation in the colouring of the residues.

To assess the variability of each predicted site in terms of the residues that line the site we employed Provar [26] a method recently developed in our group for the calculation and depiction of surface cleft variability. Provar uses an ensemble of conformers and their predicted pockets as input, calculates the propensity of each residue to line a pocket, and aids visualization by mapping the results on a single conformer structure. Provar results for the 100 CONCOORD conformers of A1AT are summarized in Figure 4. The Provar analysis is consistent with SiteMap analysis data (Figure 3B), and provides additional information about which residues are consistently part of a pocket and which are only occasionally so. For example, the majority of the residues lining the A pocket appear to be persistently part of a cleft across the CONCOORD conformer ensemble (Figure 4C). By contrast, of the residues surrounding the I pocket, only three are consistently pocket-lining: Leu276, Ile375 and Lys380 (Figure 4D). As the I pocket is only identified in about a quarter of all conformers, these residues must be often part of a different pocket that incorporates part of the I site. Moreover, Provar offers an insight into how conformational changes affect a pocket: pockets that have many of their residues coloured red (e.g. site A, Figure 4C) are likely to be changing in volume (as evidenced also in Figure 3) by “breathing”-style motions that inflate and deflate the site without having much effect on which residues are pocket-lining. Sites that have many residues surrounding them coloured pink (e.g. site I) are either transiently observed, or change shape and volume by burying and exposing different parts of the site in different conformers. Such sites are consequently more likely to be missed by software that identifies pockets, if only one conformation or poor sampling of conformers is used.

### Global Fragment and DrugBank Library Docking Studies

**Figure 5:**
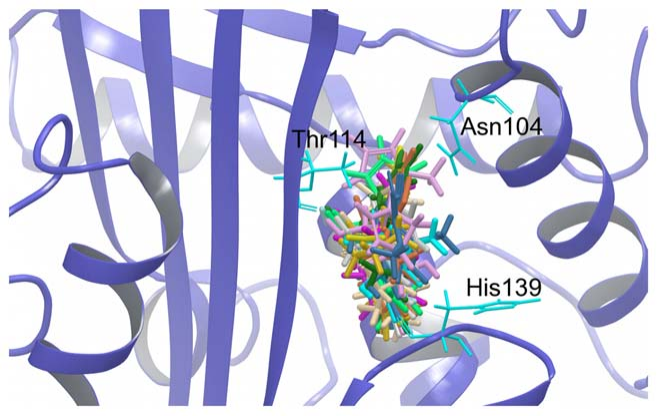
Fragment docking to the A site targets the pharmacophore defined by Asn104, Thr114, and His139. Best poses of the top-scoring 20 fragments (coloured sticks) from the ZINC dataset docked in the A site of A1AT (cartoon, blue). The majority of these fragments fill the pocket defined by Thr114 and Asn104 at the top, and His139 at the bottom (thin sticks, cyan), identified in our previous study as a potential allosteric site for targeting A1AT polymerization. Some of the fragments take advantage of hydrogen bonding opportunities presented by His139 and Thr114.

To further characterize the A–I pockets we proceeded to dock a set of representative fragments from the ZINC database and compounds from the DrugBank library to each of the sites on A1AT using Glide. Our *in silico* fragment screen identified high-scoring fragments for each site, highlighting chemotypes that may be used as starting points for future *in vitro* exploration. The ZINC identification codes for the 5 top-scoring fragments against each site are provided in Table S2. The docked poses of these fragments can be used to define pharmacophores for each site. Encouragingly, the top scoring fragments for the A site clustered in the area identified in our previous proof-of-principle study as a target for pharmacophores capable of blocking polymerization of A1AT while preserving inhibitory function (Figure 5). The area of the pharmacophore is defined by Asn104, Thr114 and His139, and several of our fragment poses favour hydrogen bonds to the threonine and histidine residues.

The protein-fragment interactions within the other, less well characterized, sites provide great insight into the ligand-binding capabilities of these pockets. For example, the top 10 fragments in the I site have at least one hydrogen bond to one of three residues: Thr273 (side-chain oxygen OG1 acts as an acceptor to 5 ligands), Lys380 (backbone oxygen O acts as donor to 7 ligands) and His269 (ND1 acts as donor to 4 ligands). Moreover two areas within the site are often occupied by hydrophobic rings. These findings can be used to build a pharmacophore template for further searches of additional ligand databases.

Overall the sites identified by SiteMap analysis demonstrated specificity even when probed with small fragment compounds, that are intrinsically more likely than larger compounds to bind promiscuously [27]. Top-scoring fragments for each site typically scored better for binding at that site than against any other site (Figure S3).

**Figure 6:**
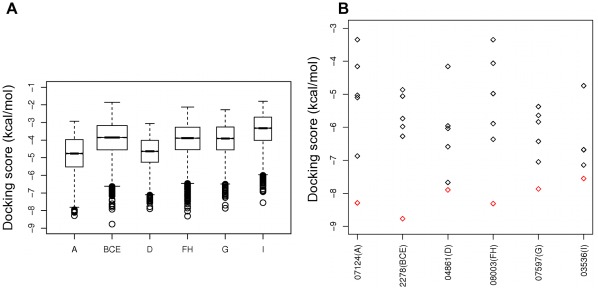
Results from docking the DrugBank collection against nine pockets on α_1_-antitrypsin. (A) Boxplot distributions of docking scores for DrugBank molecules docked to each of the nine sites A to I. Only the top-ranking pose is included for each ligand and only ligands of molecular weight less than 500 Daltons are included in this plot. (B) The best-scoring ligand for each site is assigned a worse score when docked against each of the other sites. The red diamonds represent the best docking score for each ligand depicted in Table 2, when docked to the site where it is ranked top. The black diamonds correspond to the scores for each of these ligands when docked to all other sites. The x-axis labels correspond to the DrugBank ID of the ligand and, in brackets, the site against which it is selected as “best-scoring”, e.g. 07124(A) refers to DrugBank entry DB07124 which achieves its best score against site A.

**Figure 7:**
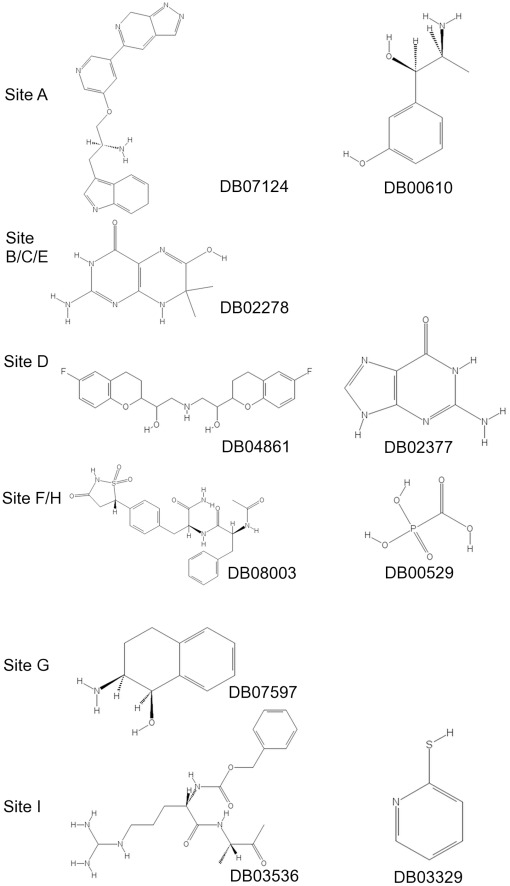
Top-scoring DrugBank molecules against the α_1_-antitrypsin sites. IUPAC names and PubChem CIDs for the DrugBank IDs in Figure 6 and Table 2 are: DB07124: 3-[(2S)-2-amino-3-[(5-{7H-pyrazolo[3,4-c]pyridin-5-yl}pyridin-3-yl)oxy]propyl]-6H-indole, PubChem CID: 46937052; DB00610: 3-[(1R,2S)-2-amino-1-hydroxypropyl]phenol, PubChem CID: 5906 DB02278: 2-amino-6-hydroxy-7,7-dimethyl-3,4,7,8-tetrahydropteridin-4-one, PubChem CID: 3340355; DB04861: 1-(6-fluoro-3,4-dihydro-2H-1-benzopyran-2-yl)-2-{[2-(6-fluoro-3,4-dihydro-2H-1-benzopyran-2-yl)-2-hydroxyethyl]amino}ethan-1-ol, PubChem CID: 71301; DB02377: 2-aminopurin-6-one, PubChem CID: 764; DB08003: (2S)-2-acetamido-N-[(2S)-1-amino-1-oxo-3-[4-[(5S)-1,1,3-trioxo-1, 2-thiazolidin-5-yl]phenyl]propan-2-yl]-3-phenylpropanamide, PubChem CID: 9547915; DB00529: phosphonoformic acid, PubChem CID: 3415; DB07597: (1R,2S)-2-amino-1,2,3,4-tetrahydronaphthalen-1-ol, PubChem CID: 6420129; DB03536: benzyl N-[(1S)-4-[(diaminomethyl)amino]-1-{[(2S)-3-oxobutan-2-yl]carbamoyl}butyl]carbamate, PubChem CID: 6398520; DB03329: pyridine-2-thiol, PubChem CID: 2723698.

Our second docking experiment scanned all pockets with the DrugBank collection of small molecules in an effort to identify any high-ranking ligands that are already used, or being tested as drugs for different targets. 12,115 small molecule ligand structures based on 5,897 molecules from the DrugBank library were docked using Glide (see Methods for details) to each of the nine surface clefts A–I. Docking scores for each ligand successfully docked to each site are summarized in Figure 6. In these plots we have merged the distribution of docking scores for sites B, C and E (labelled as site BCE), as well as F and H (FH), as the necessity of using a reasonable-size receptor grid in docking means that we cannot exclude ligands from docking to neighbouring sites, even if the grid is centred on a specific site. As is usual for docking calculations, the majority of the ligands interacted *in silico* with relatively poor predicted binding energies (−3 to −5 kcal/mol), indicating poor potential for drug development. However, promisingly, low energy outliers in these distributions achieve scores in the range of −7.5 to −8.8 kcal/mol for each site (Table 2 and Figure 7). These scores are comparable to the score of compound “CG”, a molecule identified in a previous study as an inhibitor of A1AT polymerization (CG achieves a score of −8.7 kcal/mol against its target site (A) after induced fit docking using Glide). Moreover, the best-scoring ligand for each site appeared highly selective for that site (Figure 6B). The best overall scores were achieved for sites BCE and FH. The highest-scoring ligand interaction was for 7,8-dihydro-7,7-dimethyl-6-hydroxypterin (DrugBank ID DB02278). Despite the relatively small size (209 Da) of this ligand, it achieved a score of −8.8 kcal/mol against the BCE site. However in our simulations, this molecule bound the RCL with likely adverse effects on the enzyme inhibitory function of A1AT.

**Table 2:** The best-scoring and “best-efficient” small molecules from DrugBank docked against each of the sites A-I on A1AT.

	Best overall docking score			Best scoring within ten most efficient		
Site	DrugBank ID	Glide SP score(kcal/mol)	Molecular Weight (Daltons)	DrugBank ID	Glide SP score(kcal/mol)	Molecular Weight (Daltons)
A	DB07124	−8.3	384.4	DB00610	−7.9	167.2
B/C/E	DB02278	−8.8	209.2	Same as best overall		
D	DB04861	−7.9	405.4	DB02377	−7.2	150.1
F/H	DB08003	−8.3	486.5	DB00529	−6.8	126.0
G	DB07597	−7.9	163.2	Same as best overall		
I	DB03536	−7.5	379.4	DB03329	−6.2	111.2

Diagrams, IUPAC names and PubChem CIDs for all DrugBank entries in this table can be found in Figure 7.

Since larger compounds (>350 Da) are considered unfavourable as leads for drug design we also considered the 10 best performing ligands in terms of their ligand efficiency for each site. Ligand efficiency is traditionally defined as the docking score divided by the number of heavy atoms, but here we are referring to the natural logarithm scaling of the ligand efficiency, a metric that the Schrödinger developers suggest gives a better fit to experimental data. Within these best ligand efficiency sets we then selected the ligand with the best overall docking score to avoid overcompensating for size at the expense of docking score. Some of these (‘best-efficient’) ligands conserved interactions that are important in the binding of the highest scoring ligand overall (‘best-overall’). Thus, within the I site, hydrogen bonding of a charged amine group to the backbone of Ser140 was seen with both the best-efficient (DrugBank ID: DB00610) and best-overall (DB07124) ligands. Similarly the aromatic ring of the most efficient ligand for the I site (DB03329) overlaps with the positions of all other aromatic rings in the top 10 scoring ligands.

### Induced Fit Screening for Promising I Site Ligands

For a flexible protein, like A1AT, rigid receptor docking is likely to miss many ligands that require small structural rearrangements in order to fit some of the smaller sites. In this case, docking calculations that allow for induced fit are recommended. We experimented with the induced fit protocol mostly with the I site, as this is the smallest of all and more likely to benefit from such a protocol, whereby ligands are docked into sites in a soft mode (repulsive forces are very much reduced), then the protein and the ligand are allowed to relax, and finally the ligand is redocked to the relaxed conformer of the receptor. We found that the induced fit docking protocol dramatically changes the results for some ligands.

**Figure 8:**
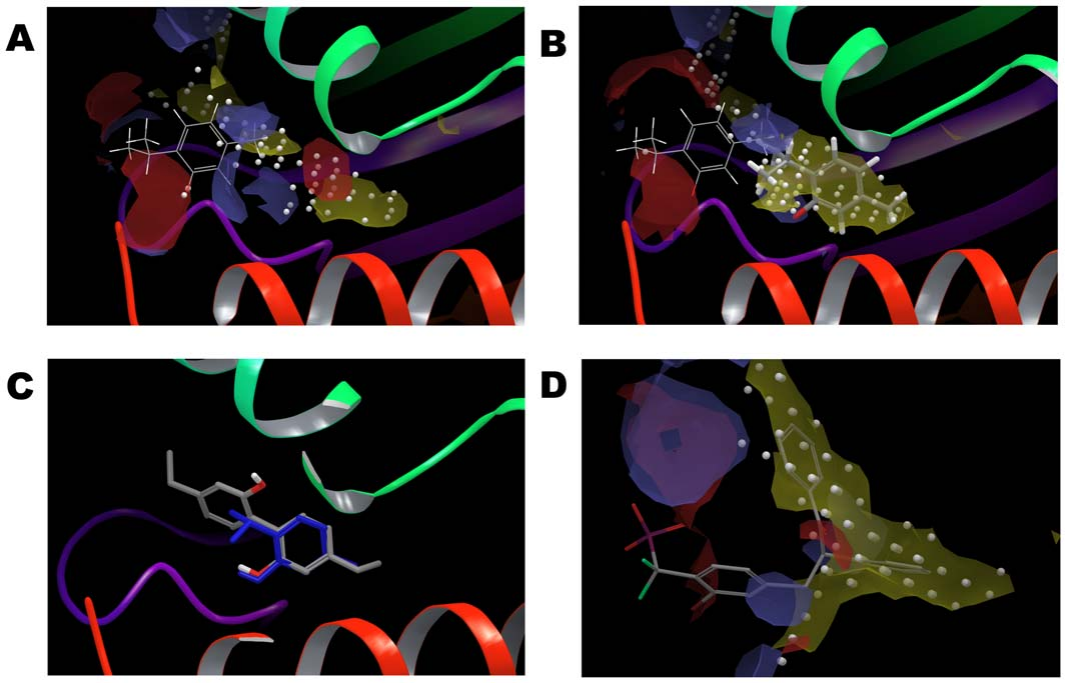
Induced fit docking allows the discovery of high affinity hits for site I. (A) Thymol (DrugBank ID DB02513, in wire representation) docks on the outside of the main cavity of the I site (small white spheres) and does not reach the hydrophobic pocket within the cavity (yellow surface), resulting in a poor docking score (−3.2 kcal/mol). (B) After induced fit docking, thymol (in stick representation) enters the site, which now comprises a larger hydrophobic cavity; the docking score is consequently greatly improved to −7.8 kcal/mol. The initial docked pose of thymol before the application of IFD is shown superimposed in wire format. (C) A derivative of thymol, 5-ethyl-2-(4-ethyl-2-hydroxyphenyl)phenol, (PubChem CID 19850961, sticks coloured by element) achieves an impressive score of −10 kcal/mol after induced fit docking, whilst retaining the original thymol pose (in blue) for the substructure that is common to both molecules. (D) Best-ranking pose for DrugBank ID DB07263 ([{2-bromo-4-[(2R)-3-oxo-2,3-diphenylpropyl]phenyl}(difluoro)methyl]phosphonic acid, in stick representation) following induced fit docking. In this protein conformer, the channel connecting sites I and C has been opened creating two hydrophobic subpockets (predicted by SiteMap and depicted here in yellow semi-transparent surface). Two of the aromatic rings of this ligand are placed in these subpockets. This ligand achieves a very good docking score (−9.5 kcal/mol), despite the fact that several hydrogen bonding opportunities (depicted by the blue and red surfaces, corresponding to H-bond donor and acceptor, respectively) are not satisfied in the case of this ligand.

We illustrate two examples here of two natural compounds with promising results. Menthol (DB000825) is a natural compound of mint oils that scores reasonably well (−6.6 kcal/mol) in the original docking trial (with the receptor kept rigid) and, more importantly, ranks eighth out of the 10,000 reported ligand poses. Following induced fit docking, this score improves dramatically to −8.5 kcal/mol, aided by a small rearrangement of His269, which results in an additional hydrogen bond to the ligand. Thymol is another interesting hit against site I. In preliminary docking experiments (without prior protein refinement in Glide) we observed that thymol was the fourth best scoring molecule against this site. Thymol is a natural product of thyme and a known protein binder [28] that is used as a stabilizer in pharmaceuticals as well as an antiseptic, vermifuge, antibiotic and fungicide, so it may be an interesting ligand to explore. Unlike many of the larger ligands that were found bound mostly on the outside of the cavity, thymol docked inside and showed a good complementarity to the site. Following protein refinement (a recommended procedure in Glide), thymol could not be docked inside the I site, resulting in a very poor docking score (Figure 8A). However, after induced fit docking thymol could enter the cavity and achieved a Glide score of −8.3 kcal/mol (Figure 8B). Finally, a series of molecules comprising the thymol scaffold resulted in several good hits, the top-scoring one being 5-ethyl-2-(4-ethyl-2-hydroxy-phenyl)phenol (PubChem CID: 19850961), which binds the I site with an impressive score of −10 kcal/mol. This score is equivalent to a K_d_ prediction in the nanomolar range (Figure 8C).

Another interesting observation relating to the I site is that there seems to be a transient hydrophobic pocket next to the originally identified pocket, which, in some conformers, is merged with that site. This can allow larger ligands with two rings connected by a flexible linker to dock in a way that takes advantage of both hydrophobic patches. For example, when docking DrugBank entry DB07263 using the induced fit protocol, we can obtain the pose depicted in Figure 8D where two of the aromatic rings are placed in the two hydrophobic subpockets making up the site in this conformer (yellow surfaces in Figure 8D). This pose achieves a very respectable Glide score of −9.5 kcal/mol. As this particular ligand does not take full advantage of the hydrogen bonding opportunities clearly depicted in the SiteMap surfaces of the site (Figure 8D surfaces in blue and red), we can assume that the affinity could be further improved by adding suitable functional groups that could interact with polar residues on the receptor.

### ThermoFluor Experiments Validate Interactions Predicted *in silico*

A small number of hits from our docking studies were assayed using thermal shift experiments (ThermoFluor). All compounds selected for testing had shown promising docking scores either using the induced fit protocol, or in preliminary docking studies using rigid receptor docking. Table 3 summarises the results for the three of the eight ligands tested that demonstrated significant thermal shifts (4-nitrocatechol, 2,6-diisopropylphenol and thymol), compared with the control substance (DMSO). The corresponding ThermoFluor graphs for these three ligands can be found in Figure S4; they show the difference between the assayed melting temperature of the incubation with compound and the average melting temperature of an appropriate A1AT control incubated under the same conditions (in this case, DMSO concentration). One of the eight compounds assayed (4-nitrocatechol, predicted to bind at the I site) demonstrated an average thermal shift exceeding 1°C. Interestingly, two of the compounds representing hits to the I site (thymol, and 2,6-diisopropylphenol) appeared to destabilise the protein, causing a negative shift in the melting temperature, which was particularly pronounced for thymol (average of −1.66°C). Negative shifts may also be due to the hydrophobic nature of the compounds, which under the assay conditions may induce non-specific destabilization of the folded state [29]. Whether stabilizing or destabilizing, the observed shifts in the melting temperature of A1AT support our docking results and indicate that the compounds assayed are most likely interacting with our protein.

**Table 3:** Shifts in melting temperature of A1AT in the presence of selected small molecule ligands (ThermoFluor assay).

Molecule Name	DrugBank ID	Average Thermal Shift in °C(*p*-value)	Predicted site of binding	Best Glide SP score after IFD (kcal/mol)
Thymol	DB02513	−1.66 (0.0089)	I	−7.8
4-Nitrocatechol	DB03407	1.92 (0.0002)	I	−6.9
2,6-Diisopropylphenol	DB00818	−1.21 (0.0021)	I	−8.3

The quoted *p*-values are the result of a Welch two-sample t-test (performed using the R statistical software) testing the null hypothesis that the difference in the mean values of the distribution of the thermal shift values for DMSO and the distribution of the thermal shift values observed for each ligand is zero. The null hypothesis was rejected for *p*-values <0.01.

## Discussion

Transiently druggable pockets on the surface of proteins can be missed by *in silico* screens to identify the most promising target site on a protein, commonly based upon a single structural snapshot. Such pockets are of particular interest in cases where the protein target undergoes large conformational variations, as in the archetypal serpin A1AT. Here, we present an alternative methodology that characterizes more pockets, and simulates their solution behaviour in greater detail than a single conformer/single pocket approach.

In this study we focused our efforts on identifying druggable pockets on the surface of native A1AT that could be the targets of inhibitors blocking polymerization. Previous *in silico* attempts to identify small molecules that can act as inhibitors of polymerisation have concentrated on what we refer to in this paper as the A site, a large cavity between the ß-sheet A and the D-helix [30]. This site was seen as a good drug target, as the space filling Thr114Phe mutation situated in the A site reduces polymerisation and preserves inhibitory function of native wild type A1AT *in vitro*, and increases secretion in a mammalian cell model of disease [31], [32]. Drug design studies based on the Thr114Phe mutant and *in silico* research focusing on this site have led to ligands that blocked polymerisation of A1AT *in vitro* [33]. However, they did so irreversibly and with the undesirable side effect of blocking the inhibitory action of A1AT [32], [34]. Hence, there is both scope and need for targeting alternative sites on A1AT. A recent attempt at identifying such sites across a range of serpins has revealed at least one site where selected sugars and amino acid derivatives may bind, acting as chemical chaperones that reduce polymerization [35]. The aim of the study described here was to identify potentially druggable sites on A1AT that have not yet been targeted in *in silico* screens.

We expected to see clues of the existence of alternative potentially druggable sites in the available crystal structures of A1AT. Indeed, crystal structures of A1AT allow us a glimpse of the variety of conformations sampled by this protein. This inherent flexibility, intimately linked to function, is dispersed across the whole protein [22], [36], [37], [38], [39] and thus potentially reflected in the properties of pockets on the surface. Analysis of available crystal structures revealed considerable variability in the surface clefts between different conformers, and suggested that this variability should not be ignored in structure-based drug design. We showed that probing deeper into the variability of potential druggable pockets could be done with a relatively cheap, constraints-based computer simulation that efficiently explores part of the protein conformational space. Additionally, we demonstrated that this approach can lead to identification of both novel (transient) sites, and also pre-existing pockets deemed non-druggable in a single crystal structure, that may demonstrate substantial druggability in the solution ensemble. Identification of such sites is the first step towards a structure-based drug design strategy that would seek to stabilize conformations where these sites are present and druggable. Such an approach may be particularly fruitful in proteins like A1AT, where the design of small molecule modulators has to strike a delicate balance between stabilizing the stressed state in order to reduce the protein’s tendency to polymerise, and preserving the protein’s antiprotease function.

The variability of each pocket was further probed using Provar, a method recently developed in our lab. Provar allows us to highlight and readily visualize residues responsible for the variability of a protein pocket across an ensemble of conformers. This information allows us an insight into the origin of the pocket variability and can assist *in silico* induced-fit type screening within high throughput studies, where keeping the number of residues that are allowed flexibility small is necessarily limited. We believe the combination of druggability and variability predictions could be very interesting for many proteins that are difficult targets due to their flexibility. We are currently pursuing an automated combination of these predictions in our laboratory.

The conformers in which each pocket achieved its highest druggability score were selected for docking studies, employing the publicly available database of marketed and experimental drugs DrugBank. These docking experiments highlighted several low molecular weight ligands that scored well on individual sites and were specific for these sites. Promisingly, several of the docking scores of our best-scoring ligands at the novel targets are comparable to the docking score of compound “CG”, a molecule previously identified as an inhibitor of A1AT polymerization *in vitro* and in mammalian cells [34].

Our approach has also revealed sites that could become the focus of future *in vitro* studies. A small but very hydrophobic site (site I) that is present in about one fifth of our *in silico*-produced conformers was initially identified by SiteMap in three crystal structures, carrying the Ala70Gly mutation. This mutation is known to increase the stability of the stressed state, oppose the propensity to polymerisation and retain the functionality of the protein, while inducing widespread changes in cavity sizes within A1AT. Further analysis showed that this site is present in all other crystallographic structures but it is not solvent accessible. Crucially it became solvent accessible in about one fifth of our conformers generated *in silico* from the wild type native structure 1qlp, indicating that transient solvent accessibility may be feasible in solution in the absence of mutations. Site I is therefore a potential ligand target site with some characteristics suggesting that ligand binding might induce local, stabilising conformational change. Support for this idea comes from mutagenesis studies that showed 13 mutations in the region of the I site (e.g. the space-filling mutation His269Tyr) increased stability, while preserving inhibitory function [22]. Thymol and menthol are both small, hydrophobic natural products that showed high complementarity to the I site, and are considered safe for use in the pharmaceutical industry. Following induced fit docking they achieved scores comparable to the score for the CG compound discovered in earlier studies. Some thymol derivatives achieved even better results, although the effect of their binding could be destabilizing, as suggested by our preliminary ThermoFluor experiments.

The channel of interconnecting sites B,C and E is also potentially interesting as the druggability scores for these pockets are persistently high, and some of our best docking scores are results of docking ligands to these sites. However, the obvious caveat of docking to this site is that many of the ligands will interact with the RCL loop, thus potentially interfering with A1AT’s antiprotease function. Indeed, mutation experiments have shown that the sequence between Arg196 to Glu279 can carry 9 mutations that increase the stability of the A1AT, but in several cases also decrease functional activity [38]. Some of the other sites explored in our study may be more promising in terms of their position on the surface and their lower potential to affect inhibitory function.

There are obvious caveats in the approach presented here. The conformers generated using CONCOORD may be artificially produced but appeared realistic when assessed for geometry and packing by the structure validation server PROSESS [25]. Moreover, the range of cavity characteristics observed was consistent with the variation between crystal structures. Although the conformational space of the protein is unlikely to be fully explored using CONCOORD, this technique did identify interesting pocket variations. In future work we plan to use the program tCONCOORD, considered better for exploration of larger variations in molecular structure [13]. The definition of pockets on the protein surface can vary significantly between programs, thus results presented here are specific to SiteMap predictions. Similarly the calculation of pocket volume and other properties are very much dependent on the definition of pocket boundaries, which varies widely across different software. Calculations of druggability are empirical, based upon previous correlations of scoring function predictions with *in vitro* observations of drug-like ligand binding. They do not guarantee *in vitro* binding affinity in a new system but provide a reasonable starting point for docking studies *in silico* and *in vitro*. Finally, our docking calculations are subject to many approximations. They should therefore be considered as a screening tool, based upon goodness of fit of certain ligands against each site, to enrich true positive hits among the ligand rankings.

In summary, we have presented a promising strategy that utilizes multiple protein conformer structures to identify both persistent and transiently druggable surface pockets. We have applied this approach to A1AT, whose conformational flexibility suggests that the usual one conformer/one pocket approach to screening is likely to be inadequate. We have found that pockets on the surface of A1AT show considerable variability across conformers, and we have also identified a new transient pocket with druggability potential. Some of our docking hits to this and other sites are at least as good or better than a previously identified promising lead. Finally, an unusually high proportion of a limited set of our *in silico* hits targeted at the I site and assayed by ThermoFluor alter the melting temperature of A1AT. These data are consistent with an *in vitro* interaction and indicate that further experiments are warranted to pursue these ligands and I site targeting.

## Materials and Methods

### Selection of α_1_-antitrypsin Crystal Structures

A1AT structures were retrieved from the PDB using the SAS tool available in PDBsum [40]. The amino acid sequence of the structure with PDB id 1qlp was used to search the PDB, and all sequences with percentage sequence identity higher than 97% were kept (this very high cut-off was used as we were only interested in sequences that did not deviate significantly from the wild type A1AT). Among identical sequences representing identical states, the highest resolution available was kept. Structures with cleaved chains, where the break in the chain was not in the RCL were removed. Our final dataset (summarised in Table 1) comprised structures that sampled different features, such as the stressed and relaxed forms, point mutations, and ligands that induce stability. More specifically, there are six native stressed and two relaxed A1AT structures, all with resolution better than or equal to 2.6 Å. The six native stressed structures can be separated into two groups. The first group comprises PDB entries 1qlp [30], 2qug [41]and 3cwm [41], which have no mutations and share nearly 100% sequence similarity (except for minor variations in the length of the C- and/or N-terminus). A partially stabilising ligand, citrate, is present in 3cwm. The second group comprises 1hp7 [42], 1oph [43] and 3drm [32], all representing the native stressed fold but with partially stabilising mutations in the sequence. Finally, of the two relaxed structures, one is an uncleaved kinetic trap of A1AT (1iz2 [37]) with ten mutations, and the other is a cleaved form, with no mutations in its sequence, and co-crystallised with the substrate (1ezx [19]).

### Identification of Surface Pockets and Calculation of their Properties

One protein chain from each crystal structure in our dataset was prepared using the Protein Preparation Wizard protocol available in the Schrödinger suite (Maestro package version 9.0 from Schrödinger, LLC). Ligands, waters and other co-crystallised agents were deleted and hydrogen atoms were added. The protassign script was used to optimise intramolecular contacts. The impref script was used to perform a restrained minimisation of the protein, with a maximum root mean square deviation (RMSD) of 0.30 Å.

All structures were superimposed on the native wild type protein (1qlp) using the structalign utility from Schrödinger. The site recognition software SiteMap 2.3 (Maestro package version 9.0 from Schrödinger, LLC) was run on all 8 crystal structures to identify the top 10 ranked potential ligand-binding sites. SiteMap uses an algorithm analogous to the Goodford’s GRID algorithm [44], which uses interaction energies between the protein and grid probes to locate energetically favourable sites. Sites were kept if they comprised at least 15 site points. A restrictive hydrophobicity definition, a standard grid (1.0 Å) and the OPLS2005 force field were used (default settings in SiteMap 2.3).

The following physicochemical properties of the sites were calculated by the SiteMap program: size, volume, degree of enclosure/exposure, degree of contact, hydrophobic/-philic character, hydrophobic/-philic balance and hydrogen-bonding possibilities (acceptors/donors). In addition, SiteMap calculates two scores for each site. The SiteScore is defined as:
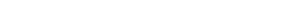
where

*n*  =  the number of site points (capped at 100),

*e*  =  enclosure,

*p*  =  hydrophilicity of the site (capped at 1.0).

The druggability score, Dscore, is defined as:
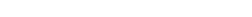
where *n*, *e* and *p* are defined as above, except that *p* is uncapped in this case. The developers of SiteMap suggest that a cut-off in the SiteScore of 0.80 can be used to differentiate between drug-binding and non-drug-binding sites, with scores higher than 1.0 being indicative of highly promising sites [45]. The Dscore can help to distinguish between undruggable and druggable sites, by penalising highly hydrophilic sites, as ligands binding to such sites would be very polar, and would be quickly eliminated by the organism. This does not mean that the site cannot bind any ligands, but that it would be difficult to find high affinity drug-like ligands for such a site [2].

Nine sites were identified in our dataset of crystal structures of A1AT. These sites were labelled A to I. The geometric centre of each site as seen in the native wild type protein (1qlp), or, in the case of the I site, as seen in the structures bearing the Ala70 to Gly mutation (1oph, 1iz2 and 1hp7), was calculated and it was used to identify sites in all other crystal structures and computationally produced conformers. This was done as follows: If the geometric centre of a site *k* was within 3.75 Å of the geometric centre of any site *s* (where *s* ∈ {A,B,C,D,E,F,G,H,I}) then site *k* was assigned the letter of the site *s* (i.e. the two sites were thought to coincide). This cut-off is strict and it was chosen after manual inspection of several cases where sites were very close to each other, but where it was still possible to discriminate between them. Sites C and E overlap in many conformers and in these cases they were assigned the label “C_E”. If the calculated distances for a new site were between 3.75 and 10 Å, the sites were inspected and assigned manually. If all distances were above 10 Å, the site was categorised as being new. Inspection of all “new” sites found in conformers of 1qlp led to approximately half of these sites being reassigned to one of the original nine sites (A to I). The remaining unassigned sites included mostly low-scoring sites, which were ignored in the present analysis.

### Generation of Protein Conformers Using CONCOORD

CONCOORD 2.0 [24] was used to produce alternative conformations for the native wild type proteins (1qlp and 2qug). The input structures were prepared with Schrödinger’s Protein PreparationWizard, as detailed above. CONCOORD builds a library of distance constraints based on the observed interatomic distances in the original structure. Interactions deemed to be stronger are given tighter constraints. The program then produces randomly a large number of potential conformations, and attempts to correct structures with atom-pair distances falling outside the allowed regions. We allowed 1000 iterations of the correction algorithm per structure, and rejected structures whose interatomic distances violated the original distances by more than 3nm in total. We set CONCOORD to an output of 100 novel conformations for the native wild type proteins (1qlp and 2qug), which fulfilled the distance constraints. The maximum RMSD from the original structure was 2.96 Å. We have also performed a CONCOORD run that produced 5000 conformers based on the 1qlp structure. These were only used for comparison to our more limited 100 runs. All computationally produced conformers were superimposed on the native wild type (1qlp) using the structalign program.

We have evaluated the quality of the CONCOORD conformers using the PROSESS server [46] available at http://prosess.ca. Table S1 contains a summary of these results.

### Automated Assessment and Visualisation of Surface Pocket Variability

For each of the 100 CONCOORD conformers potential druggable sites were identified using SiteMap 2.3, as detailed previously. The SiteMap output files were then merged into single PDB files containing all predicted site sphere coordinates and were used as input to our in-house pocket variability visualisation method Provar. The Provar method is explained briefly here: For each conformation, residues within 3.75Å of any SiteMap sphere were considered as being pocket-lining and assigned a score of 1, all other residues were assigned a score of 0. These scores were summed across all conformations and divided by the number of conformers (100) to assign each residue a probability value representing the likelihood that it borders a predicted site. These values were written to the B-factor column of the PDB file (1qlp), and results were displayed using Chimera. Residue atoms and ribbons were rendered on a continuous colour scale from white (low probability set to the value of the first quartile of the distribution) to red (high probability set to the value of the third quartile).

### Docking

Each of the nine sites (A to I) was used as a target for docking small molecules. For each site, the CONCOORD conformer that was selected to dock to was the one with the highest volume among the ones with the top five SiteScores as predicted by SiteMap. This selection was justified on the grounds that the highest SiteScore was not always associated with the largest cavity, but in rigid receptor docking a larger cavity, which allows more room for ligands to bind can potentially make up for the lack of side-chain flexibility during docking. Receptor grids were calculated with Glide (Maestro package version 9.0 from Schrödinger, LLC), keeping default settings. The grid box was centred on the calculated geometric mean of the particular site. The box side lengths were set to the maximum value (14 Å).

All ligand libraries used in this study were prepared using LigPrep (Maestro package version 9.0 from Schrödinger, LLC). The preparation involved the generation of up to 32 stereoisomers (where these were not defined), tautomers, and protonation states corresponding to a pH of 7±2 (using epik), as well as an energy minimisation of the 3D structure using the OPLS2005 force field. The DrugBank 3.0 [47], [48] library comprised 5897 entries after filtering to remove entries larger than 500 Daltons. Following LigPrep preparation, this library consisted of 12115 small molecules. The library referred to as “ZINC fragments” is a representative library of fragments, based on the 3632 ZINC “clean fragments” subset clustered at the 60% Tanimoto similarity (downloaded from ZINC on the 05/06/2011). The clean fragments dataset obeyed the following criteria: xlogP< = 2.5, molecular weight < = 250 Daltons and number of rotatable bonds < = 5. Following LigPrep preparation, this library contained 5324 small molecules. Finally, a small subset of the PubChem library (1326 ligands related to thymol and extracted from PubChem, using the “Similar Compounds Search” on the web entry for thymol) was also prepared using LigPrep.

Glide with standard precision (SP) scoring was used for docking. Epik 2.0 state penalties were used in the final scoring. The highest scoring pose per ligand was kept and post-docking minimisation was switched on.

#### Induced fit docking

A small number of molecules were selected for induced fit docking (IFD). All these molecules had shown promising Glide SP scores in preliminary docking trials, but some had poor scores following the inclusion of a protein preparation step. This suggested that IFD might be able to restore or even improve on the original scores, as it allows the protein side chains to optimise their position in the presence of the ligand. The IFD protocol (Maestro package version 9.0 from Schrödinger, LLC) available within the Schrödinger suite was employed [49]. Briefly, this protocol involves docking the ligand using a softened potential, and refining selected docked poses using Prime side-chain prediction and minimisation [49]. The refined protein conformations are then used for the final Glide docking step, where ligands are redocked, keeping the protein rigid. Default values were used for all Glide and Prime parameters. As the protein was prepared in advance no additional refinement was performed at this stage. For the initial Glide docking both the receptor and ligand van der Waals scaling were set to 0.50. Up to 20 poses were kept. The Prime induced fit step refined residues within 5.0 Å of the ligand poses by optimising their side chains. In the final step, the ligand poses were redocked using Glide SP into structures within 30.0 kcal.mol^−1^ of the top 20 structures.

We applied the IFD protocol to a small selection of ligands docked in the I and C sites. This procedure was also applied to dock the CG compound [34] to the A site in the native wild type A1AT (1qlp).

### Figures

All figures depicting A1AT, except Figure 8, were created using the UCSF Chimera [50] package from the Resource for Biocomputing, Visualization, and Informatics at the University of California, San Francisco. Figure 8 was created using Maestro. The small molecule 2-D diagrams of Figure 7 were created using the CACTVS editor csed [51]. Plots in Figures 3B-F and 6 were created using the statistical software R (available at http://cran.r-project.org/doc/FAQ/R-FAQ.html).

### ThermoFluor Studies

The ThermoFluor (thermal shift) assay was performed using the iQ5 Real Time detection System (Bio-Rad – PCR Machine). Protein unfolding was monitored by measuring the fluorescence of the solvatochromic fluorescent dye SYPRO Orange, signalling unfolding of the protein. The compounds to be screened were dissolved in 100% dimethyl sulfoxide (DMSO) to give a stock solution of 20 mM. The assay was performed in 96- well plates, each well totalling a volume of 25 µL. Every assay had a final concentration of 1 mg.mL^−1^ of A1AT, 1 mM of compound giving a final DMSO concentration of 5%, to which 1 µL SYPRO Orange (1:200 dilution) was added. Furthermore, the influence of DMSO and A1AT concentration on the thermal shift were analysed. The DMSO concentration was varied to 5%, 10% and 15% and the concentration of A1AT from 1 mg.mL^−1^ to 5 mg.mL^−1^. Each trial was repeated 6 times (except the 5 mg.mL^−1^ concentration of A1AT, n = 2). The starting temperature for each run was 10°C increasing to 95°C in 0.5°C steps.

## Supporting Information

Figure S1 **Exploration of conformational space of A1AT using CONCOORD.** CONCOORD-generated conformers from a native wild type A1AT structure ((PDB: 1qlp). (A) All 100 conformers used to analyse druggability of sites and their occurrence. (B) The 7 structures used for docking to sites A–I; colours for conformers are: white (site G), magenta (sites E and F), cyan (site I), yellow (sites A and C), red (site B), blue (site H), green (site D). (C) Three selected conformers depicting the extent to which structural variation was simulated.
(TIF)

Figure S2 **A channel of interconnecting pockets on the surface of A1AT.** (A) A channel of interconnecting surface pockets (blue spheres) defined by the RCL at the top and the H-helix at the bottom can be seen in several *in silico* produced A1AT conformers. (B) This channel is split up into separate sites in most conformers: B (cyan), E (fuchsia), I (yellow). These subsites themselves occasionally overlap as in the case shown here, e.g. site E can “spill into” the spaces usually occupied by sites I and B.
(TIF)

Figure S3 **Site specificity of high-scoring fragment molecules.** Red diamonds represent the docking scores for the top 5 scoring fragments for each of the sites A, BCE, D, FH, G, and I. The boxplots summarise the corresponding (merged) distributions of docking scores for the same five fragments docked to all other sites.
(TIF)

Figure S4 **Thermal shift and melting temperature assays for A1AT incubated with selected ligands.** Fluorescence-based (Thermofluor) thermal shift assay curves for A1AT incubated with small molecule ligands. Only ligands with significant thermal shifts are shown. Representative curves obtained in the presence of these ligands (solubilised in DMSO, final concentration 5% (v/v)) are shown in plots A to D (control with 5% DMSO in grey, data from incubation with ligands in red). The mean ΔT_m_ is shown for A1AT incubated with each ligand: (A) 5% DMSO control, (B) 4-nitrocatechol, (C) 2,6-diisopropylphenol, (D) thymol. (E) Mean melting temperatures and standard deviations for A1AT incubated with these three ligands (red) or 5% DMSO control (grey).
(TIF)

Table S1 **Overall quality results for crystal structures and** ***in silico*** **conformers of A1AT selected for docking assessed by the PROSESS server (** http://prossess.ca **).**
(DOC)

Table S2 **Results for top-ranking fragments against each of the sites A–I on A1AT.** The ZINC molecule identification codes and Glide SP docking score (within brackets, in kcal/mol) for each of the five top-ranking fragments docked to sites on A1AT are listed. Results for sites B, C, E and F, H are merged.
(DOC)
