# Supplementary figures and images for: In Silico Assessment of Potential Druggable Pockets on the Surface of α1-Antitrypsin Conformers
Source: PLoS One. 2012 May 8;7(5):e36612. doi: 10.1371/journal.pone.0036612 (PMC3348131; doi:10.1371/journal.pone.0036612)

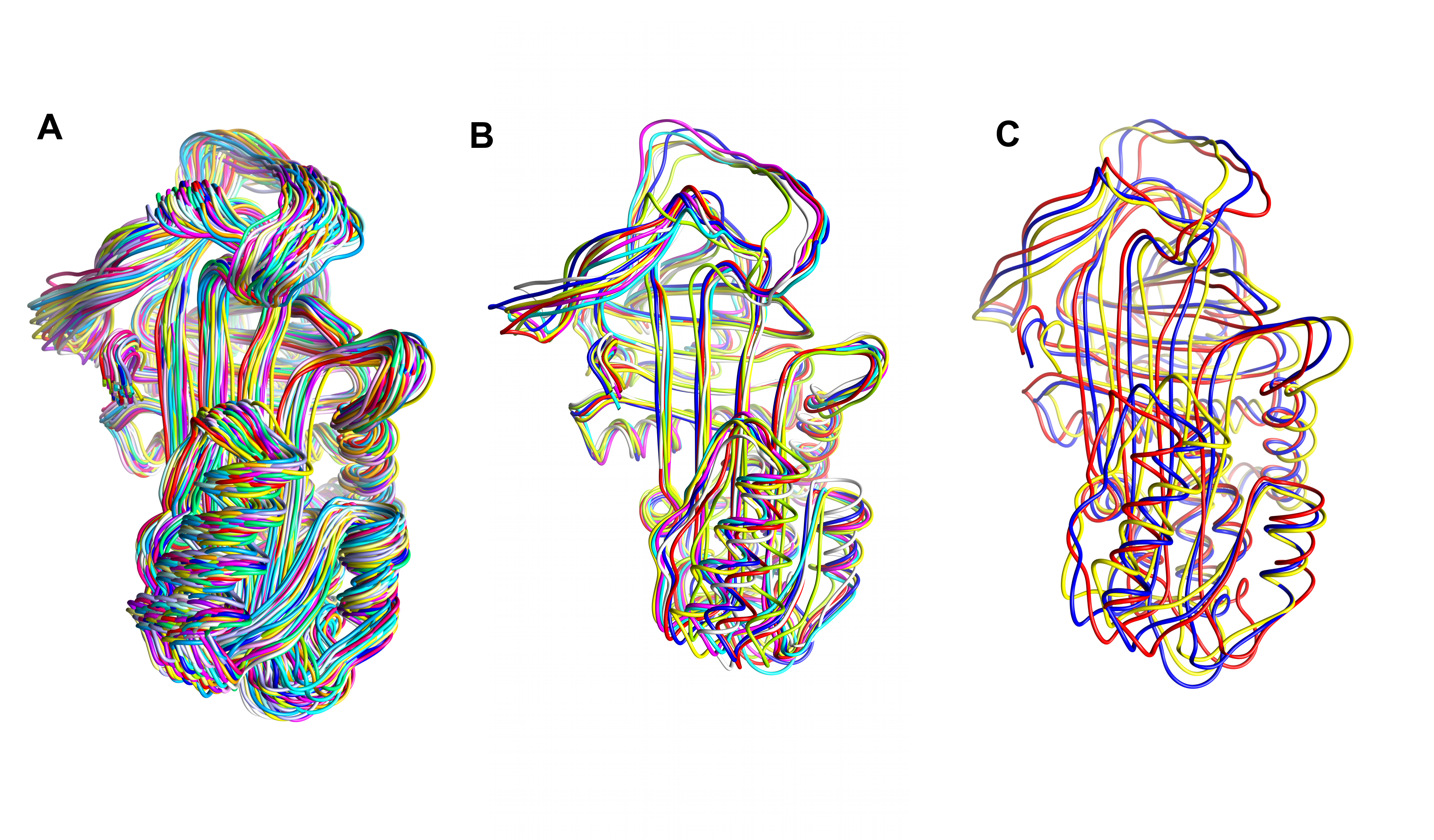

Supplement: Figure S1 — Exploration of conformational space of A1AT using CONCOORD. CONCOORD-generated conformers from a native wild type A1AT structure ((PDB: 1qlp). (A) All 100 conformers used to analyse druggability of sites and their occurrence. (B) The 7 structures used for docking to sites A–I; colours for conformers are: white (site G), magenta (sites E and F), cyan (site I), yellow (sites A and C), red (site B), blue (site H), green (site D). (C) Three selected conformers depicting the extent to which structural variation was simulated. (TIF) [file pone.0036612.s001.tif]

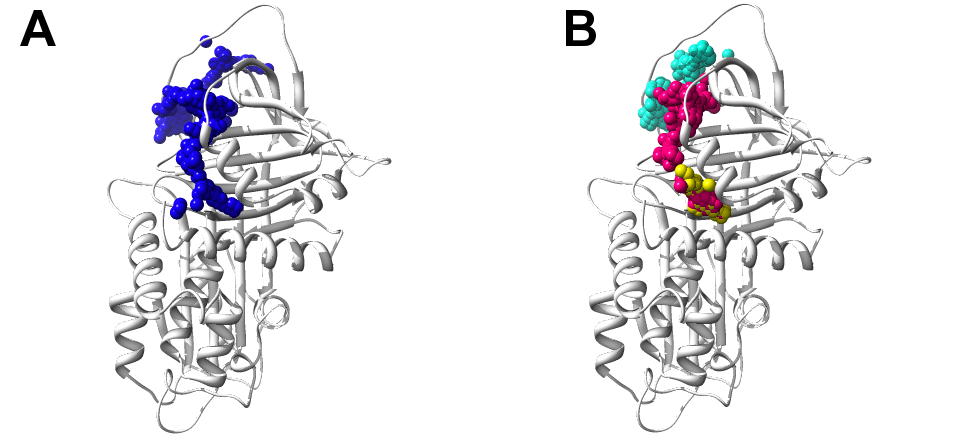

Supplement: Figure S2 — A channel of interconnecting pockets on the surface of A1AT. (A) A channel of interconnecting surface pockets (blue spheres) defined by the RCL at the top and the H-helix at the bottom can be seen in several in silico produced A1AT conformers. (B) This channel is split up into separate sites in most conformers: B (cyan), E (fuchsia), I (yellow). These subsites themselves occasionally overlap as in the case shown here, e.g. site E can “spill into” the spaces usually occupied by sites I and B. (TIF) [file pone.0036612.s002.tif]

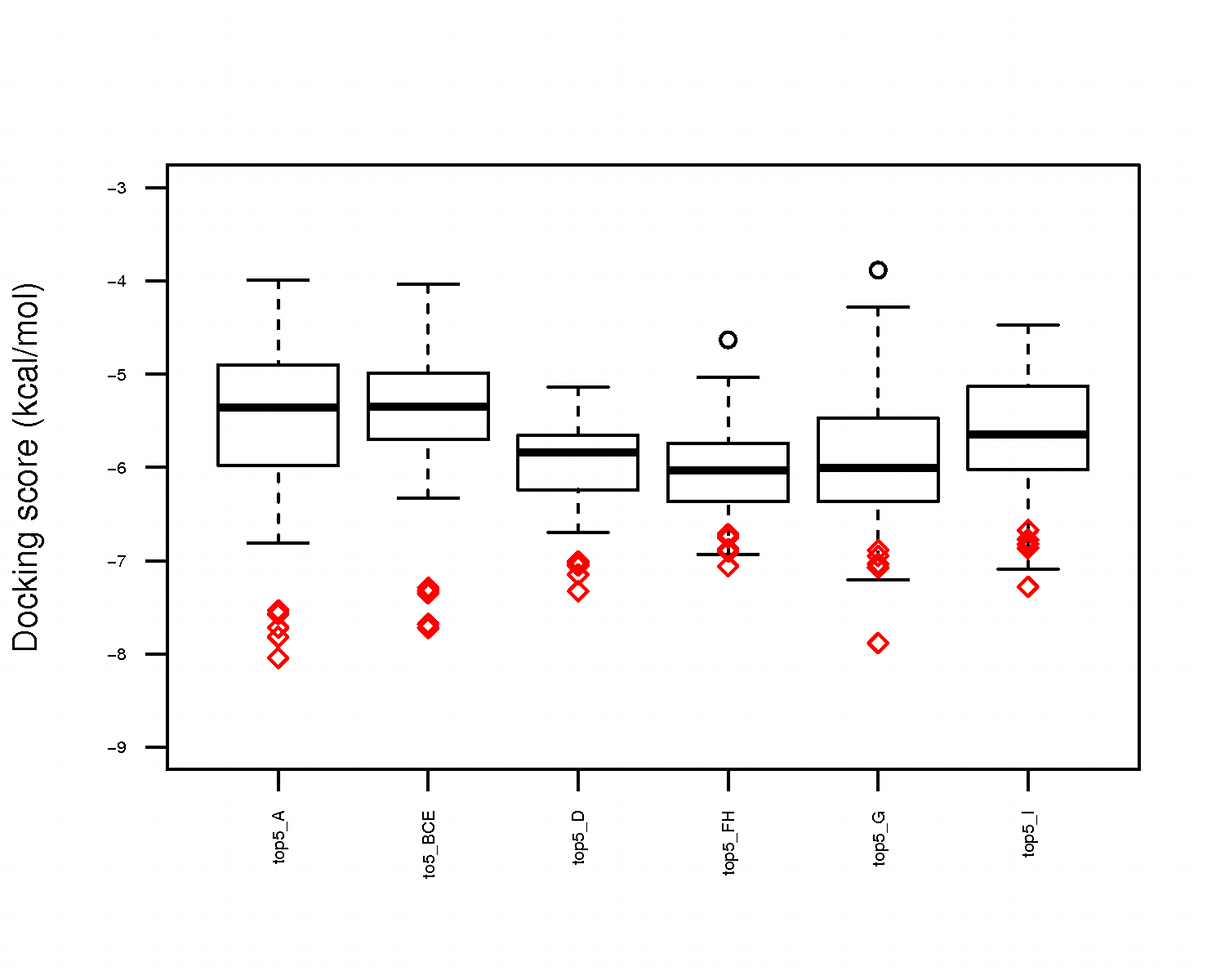

Supplement: Figure S3 — Site specificity of high-scoring fragment molecules. Red diamonds represent the docking scores for the top 5 scoring fragments for each of the sites A, BCE, D, FH, G, and I. The boxplots summarise the corresponding (merged) distributions of docking scores for the same five fragments docked to all other sites. (TIF) [file pone.0036612.s003.tif]

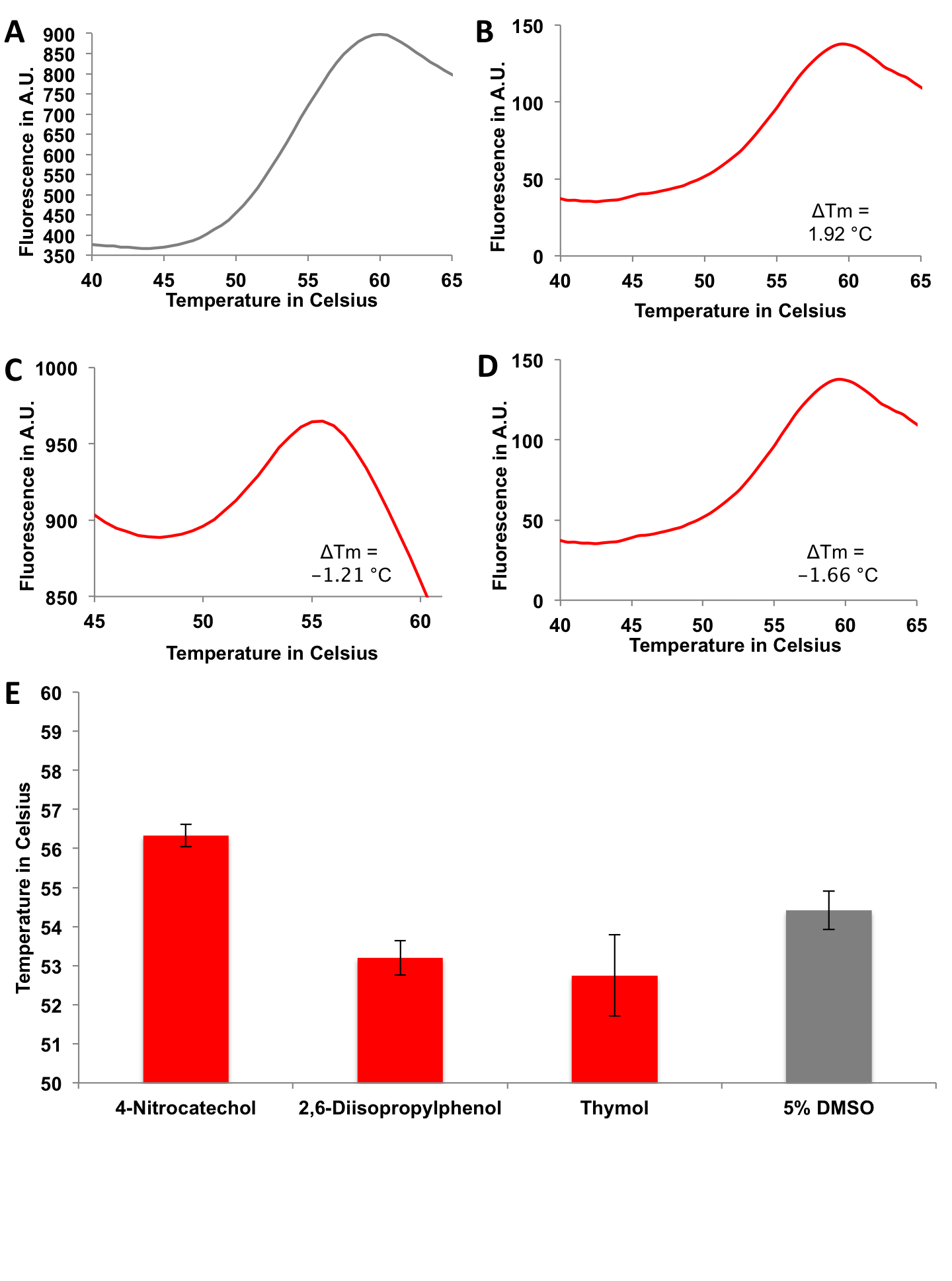

Supplement: Figure S4 — Thermal shift and melting temperature assays for A1AT incubated with selected ligands. Fluorescence-based (Thermofluor) thermal shift assay curves for A1AT incubated with small molecule ligands. Only ligands with significant thermal shifts are shown. Representative curves obtained in the presence of these ligands (solubilised in DMSO, final concentration 5% (v/v)) are shown in plots A to D (control with 5% DMSO in grey, data from incubation with ligands in red). The mean ΔTm is shown for A1AT incubated with each ligand: (A) 5% DMSO control, (B) 4-nitrocatechol, (C) 2,6-diisopropylphenol, (D) thymol. (E) Mean melting temperatures and standard deviations for A1AT incubated with these three ligands (red) or 5% DMSO control (grey). (TIF) [file pone.0036612.s004.tif]
