# Supplementary material for: In Silico Assessment of Potential Druggable Pockets on the Surface of α1-Antitrypsin Conformers
Source: PLoS One. 2012 May 8;7(5):e36612. doi: 10.1371/journal.pone.0036612 (PMC3348131; doi:10.1371/journal.pone.0036612)
Supplement: Table S1 — Overall quality results for crystal structures and in silico conformers of A1AT selected for docking assessed by the PROSESS server ( http://prossess.ca ). (DOC) [file pone.0036612.s005.doc]

**Supporting Information**

**Table S1**

| **PDB id / conformer id** | **Overall quality score** | **Covalent bond quality** | **Non-covalent/ packing quality** | **Torsion angle quality** |
| --- | --- | --- | --- | --- |
| 1qlp | 9.5 | 7.5 | 7.5 | 8.5 |
| 2qug | 1.5 | 7.5 | 7.5 | 5.5 |
| 3cwm | 7.5 | 7.5 | 7.5 | 7.5 |
| 3drm | 9.5 | 7.5 | 8.5 | 7.5 |
| 1oph | 9.5 | 7.5 | 8.5 | 7.5 |
| 1iz2 | 9.5 | 7.5 | 7.5 | 7.5 |
| 1ezx | 9.5 | 7.5 | 8.5 | 7.5 |
| Conf_77 (used for sites A, C) | 6.5 | 7.5 | 7.5 | 7.5 |
| Conf_85  (used for site B) | 7.5 | 7.5 | 7.5 | 7.5 |
| Conf_95 (used for site D) | 6.5 | 7.5 | 6.5 | 7.5 |
| Conf_53 (used for sites E, F) | 6.5 | 7.5 | 7.5 | 7.5 |
| Conf_20 (used for site G) | 7.5 | 7.5 | 7.5 | 7.5 |
| Conf_87 (used for site H) | 7.5 | 7.5 | 7.5 | 7.5 |
| Conf_57 (used for site I) | 7.5 | 7.5 | 6.5 | 8.5 |
