# Supplementary material for: In Silico Assessment of Potential Druggable Pockets on the Surface of α1-Antitrypsin Conformers
Source: PLoS One. 2012 May 8;7(5):e36612. doi: 10.1371/journal.pone.0036612 (PMC3348131; doi:10.1371/journal.pone.0036612)
Supplement: Table S2 — Results for top-ranking fragments against each of the sites A–I on A1AT. The ZINC molecule identification codes and Glide SP docking score (within brackets, in kcal/mol) for each of the five top-ranking fragments docked to sites on A1AT are listed. Results for sites B, C, E and F, H are merged. (DOC) [file pone.0036612.s006.doc]

**Supporting Information**

**Table S2**

| **Site** | **Rank 1 (score)** | **Rank 2 (score)** | **Rank 3 (score)** | **Rank 4 (score)** | **Rank 5 (score)** |
| --- | --- | --- | --- | --- | --- |
| A | ZINC01581130 (-8.0) | ZINC00347000 (-7.8) | ZINC13728763 (-7.7) | ZINC08746511 (-7.6) | ZINC57218770 (-7.5) |
| B/C/E | ZINC49587279 (-7.72) | ZINC04521093  (-7.68) | ZINC02028426 (-7.35) | ZINC13283774 (-7.32) | ZINC01645671 (-7.29) |
| D | ZINC02293661 (-7.3) | ZINC00339659 (-7.1) | ZINC17377281 (-7.0) | ZINC01559484 (-7.0) | ZINC16037356 (-7.0) |
| F/H | ZINC13217456 (-7.1) | ZINC01678957  (-6.9) | ZINC05545529  (-6.9) | ZINC05286128 (-6.7) | ZINC25949941 (-6.7) |
| G | ZINC08627928 (-7.9) | ZINC04629171 (-7.1) | ZINC14983615 (-7.0) | ZINC38701009 (-6.9) | ZINC12403009 (-6.9) |
